# Environmental Monitoring of the Littoral Zone of Lake Baikal Using a Network of Automatic Hydro-Meteorological Stations: Development and Trial Run

**DOI:** 10.3390/s21227659

**Published:** 2021-11-18

**Authors:** Mikhail Makarov, Ilya Aslamov, Ruslan Gnatovsky

**Affiliations:** Limnological Institute, Siberian Branch of the Russian Academy of Sciences, 664033 Irkutsk, Russia; ilya_aslamov@bk.ru (I.A.); gnat@lin.irk.ru (R.G.)

**Keywords:** Lake Baikal, water quality, sensors, ecological monitoring, data management

## Abstract

An automatic hydro-meteorological station (AHMS) was designed to monitor the littoral zone of Lake Baikal in areas with high anthropogenic pressure. The developed AHMS was installed near the Bolshiye Koty settlement (southern basin). This AHMS is the first experience focused on obtaining the necessary competencies for the development of a monitoring network of the Baikal natural territory. To increase the flexibility of adjustment and repeatability, we developed AHMS as a low-cost modular system. AHMS is equipped with a weather station and sensors measuring water temperature, pH, dissolved oxygen, redox potential, conductivity, chlorophyll-a, and turbidity. This article describes the main AHMS functions (hardware and software) and measures taken to ensure data quality control. We present the results of the first two periods of its operation. The data acquired during this periods have demonstrated that, to obtain accurate measurements and to detect and correct errors that were mainly due to biofouling of the sensors and calibration bias, a correlation between AHMS and laboratory studies is necessary for parameters such as pH and chlorophyll-a. The gained experience should become the basis for the further development of the monitoring network of the Baikal natural territory.

## 1. Introduction

Environmental protection and the rational use of natural resources are priorities among the fundamental scientific issues. The current ecological crisis threatens the possible sustainable development of human civilization because the further degradation of natural systems leads to the destabilization of the biosphere, as well as the loss of its integrity and ability to maintain the environmental qualities necessary for human life. Biodiversity is the most important factor in the functioning of ecosystems, and numerous studies have revealed that a decrease in species diversity in communities leads to the degradation of their ecosystem functions [1,2,3,4]. Environmental monitoring should be supported by scientific research and serve as a navigator of environmental control objects. As a rule, freshwater monitoring includes sampling and subsequent laboratory research. Samples are taken by hand from certain sites of a water body and at certain time intervals. This conventional approach provides important information about water quality and basic ecological processes. However, this approach often cannot encompass the dynamics of the many biotic and abiotic processes occurring within a smaller timescale than the sampling frequency [5,6,7,8,9]. The same is true for the monitoring of the atmosphere for CO${}_{2}$, NO, soot, and aerosols [10,11].

Baikal is not only the deepest lake and the largest drinking water reservoir, but also a unique, finely regulated ecosystem. Many natural, anthropogenic and technogenic factors affect the ecological balance of the lake: tourism, wildfires, climate change, etc. In recent years, some researchers have indicated characteristic signs of ecological crisis in the coastal zone of Lake Baikal [12]. The crisis is characterised by the massive growth in green filamentous *Spirogyra* algae and mass disease and death of endemic sponges *Lubomirskia baicalensis* (Pallas, 1771) [13]. Sponges are prevailing species of macrozoobenthos in the littoral zone, and they play a very important role in maintaining the cleanness of coastal waters. The loss of this species can be catastrophic for the ecology of this unique freshwater lake and the biodiversity of the lake ecosystem. The anthropogenic impact has led to the significant degradation of many of the Earth’s freshwater ecosystems, bringing the problem of lack of quality drinking water to the forefront, and stimulating the development and implementation of various environmental monitoring systems worldwide [14,15].

Monitoring the ecological conditions of Lake Baikal requires a system with the following functions: (i) monitoring focused on scientific instruments and modern research methods providing the quantitative evaluation of key elements of the ecological condition of Lake Baikal, including biological, hydrophysical and hydrochemical indicators; (ii) the timely development of recommendations for decision-makers concerning the sustainable use of resources of the Baikal natural territory, including economic and social fields. Therefore, it is important to develop and implement a system for automatic monitoring of the littoral zone of the lake using modern sensors that measure parameters such as pH, chlorophyll-a, dissolved oxygen, water temperature, water turbidity, and redox potential. Due to climatic conditions and the large depths of Lake Baikal, the past experience of introducing automatic stations was reduced to the installation of underwater buoy stations that recorded parameters in the internal memory (in some cases, magnetic tapes were used). Usually, these were hydrophysical buoys equipped with current speed and direction sensors and thermistor chains [16]. At that time, using autonomous hydrochemical sensors was out of the question.

Recently, with the development of technology, easy-to-use sensors that measure various parameters have become available. This has led to the emergence of many automatic monitoring system projects for freshwater bodies. There are several examples of high-frequency monitoring systems, such as those implemented at 18 reservoirs in Sardinia, which were used to determine the optimal depth of water intake and for the early identification of algal blooms [5], the monitoring system implemented at Lake Iseo, which measures the main heat, radiation and mechanical fluxes in the lake’s surface [17], and high-frequency monitoring of meteorological parameters and water temperature at different sites of Lake Como, which are used as input data for the three-dimensional hydrodynamic model [18]. A high-frequency monitoring system for the deep subalpine lakes Maggiore, Lugano and Como is developing in Italy [19]. In Russia, there is a commercial project, under which monitoring systems are being developed to monitor the flood situation. An automatic system developed and implemented by the Emercit [20] Company is Russia’s first complex monitoring system that combines all the necessary tools for a timely flood warning. Programs for the ecological monitoring of the main tributaries of Lake Baikal [21,22] and of the Angara/Yenisei river system [23], the only run-off of Lake Baikal, are also being developed. New, promising directions that allow for a significant expansion of the spatial scale of environmental monitoring are satellite monitoring [24,25,26] and sampling devices based on aerial drones [27,28] or automatic unmanned surface vehicles [29].

Considering the specifics of the climate and the steep slope of Lake Baikal bottom, we attempted to develop a monitoring station that differed from the buoy type, which could record all the necessary parameters for assessing the water quality in the littoral zone of the lake. As in other projects [30,31,32,33,34,35], we created a modular architecture, which facilitated the integration of additional sensors into the monitoring station. The data storage, transmission and final analysis on our servers allowed us to fully control the data collection system at the debugging stage, adjusting it for the necessary tasks. This study aims to provide guidelines for the establishment of a network of monitoring stations and general data quality control procedures to ensure the good functioning of the system itself and rational data management.

## 2. Materials and Methods

### 2.1. Lake Baikal, The History of the Study

**Figure 1:**
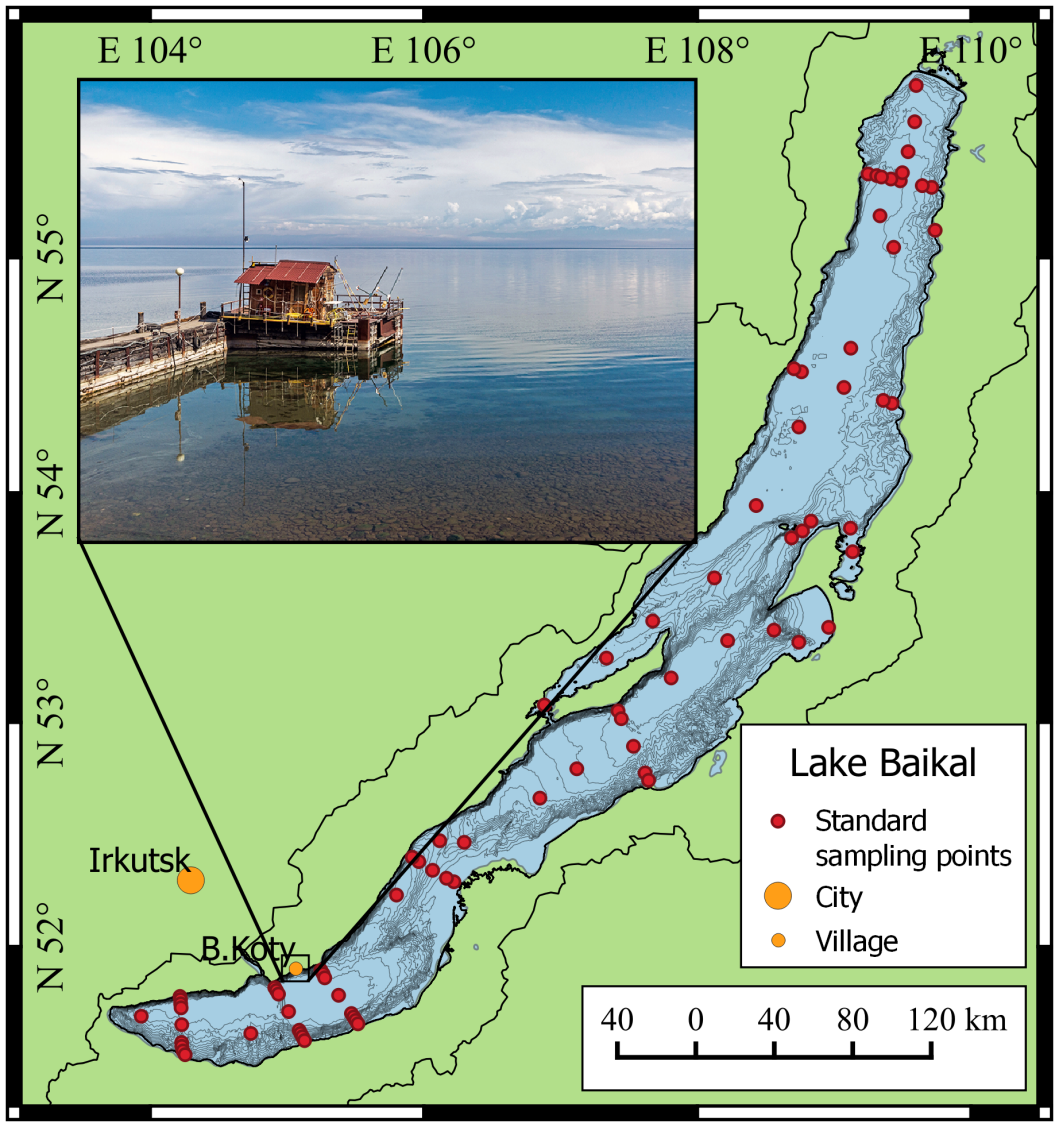
Lake Baikal and the position of the research station of Limnological Institute SB RAS. Red dots—location of standard sampling points.

Lake Baikal is located in southeast Siberia, in the central part of the deep fault of the earth’s crust between the Eurasian and Amur tectonic plates, near the Mongolian border (See Figure 1), and surrounded by mountain ranges covered with forests. Baikal is the world’s largest and deepest lake: its length is over 630 km; the average width is 60 km, and the maximum depth is over 1600 m. The total volume of fresh drinking water in the lake is 23.6 thou km${}^{3}$, which is about 20% of the volume of all surface freshwaters on the Earth. The age of the lake is approximately 25 million years. Water in Lake Baikal is characterized by extremely low mineralization values (0.95 mg/L of dry residue), high transparency (more than 40 m) and cleanness. Three hundred and thirty-six rivers flow into the lake, and only the Angara River flows out of it. The watershed area of Lake Baikal is 588.1 thou km${}^{2}$, of which about 299 thou km${}^{2}$ are located in Russia; the remaining 289 thou km${}^{2}$ are located in Mongolia, where the southern part of the Selenga River is located. The lake contains uniquely diverse flora and fauna; more than 1800 species are endemic [36]. For these reasons, Lake Baikal has attracted attention and encouraged nature enthusiasts and scientists in various fields throughout the world to recommend that the lake be included in the UNESCO World Heritage List [37].

Before the Irkutsk Hydroelectric Power Station (the Irkutsk HPS) regulated Lake Baikal water outflow, its mean water level was a height of 455.6 m above the Pacific ocean level, with annual variation of more than 1 m. The maximum was in the autumn (September–October), and the minimum was in the spring (April–May). Since 1959, after the completion of the construction of the Irkutsk HPS dam, the water level in Lake Baikal has risen by 0.8 m, exceeding the absolute value of 456.4 m. At present, the water level in the lake largely depends on the operation of both the Irkutsk HPS and the entire cascade of hydroelectric power stations on the Angara River, with usual seasonal fluctuations of less than 1 m [38].

Scientific research into Lake Baikal dates back to 1724, after the establishment of the Russian Academy of Sciences. In 1928, near the Angara River source, the Baikal Limnological Station was organized, which was later reorganized into the Limnological Institute SB RAS. At present, the Limnological Institute is the basic institution that carries out scientific research at Lake Baikal, in its tributaries and the Baikal natural territory. The institute has a fleet of research vessels equipped with modern scientific devices, such as the SBE CTD sensors, Idronaut, the Agilent Technologies laboratory complexes, etc. The research covers various fields of science: hydrophysics, hydrochemistry and microbiology. However, the studies of Lake Baikal are directed along the strictly defined grid of stations, which covers transverse and longitudinal sections as well as the coastline. Overall, there are about 110 sampling sites for the entire water area of Lake Baikal (Figure 1). At each station, sampling is carried out with subsequent laboratory research. Hydrophysical studies are also performed from the surface to the bottom.

### 2.2. Hardware of the Monitoring System: Water Sampling, Measured Parameters, Organization of Data Storage and Transmission

**Figure 2:**
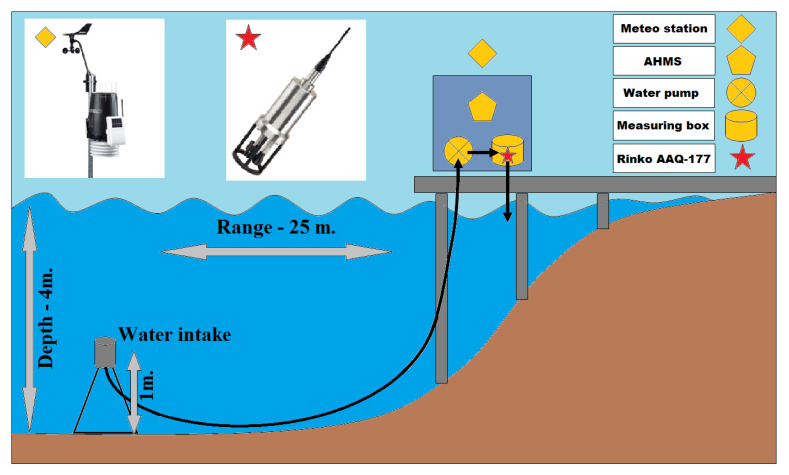
Configuration scheme and sensors of AHMS.

An automatic hydro-meteorological station (AHMS) was installed on the pier of the research station of Limnological Institute in the Bolshiye Koty settlement (latitude N 51.899651°; longitude E 105.064004${}^{{}^{\circ}}$; Figure 1). Due to the large slopes at the bottom (over 45${}^{{}^{\circ}}$) on the west coast of Lake Baikal, it was decided to organize a flow-type system to measure water parameters. Water was sampled from a 4 m depth at 25 m distance from the pier using a 32 mm polyethylene pipe and a Grundfos UPA15-90 centrifugal water pump (Figure 2). Water was supplied to a measuring box made of a 160 mm polyethylene pipe, which was vertically installed. A Rinko AAQ-177 multiparameter sensor (JFE Advantech Co. Ltd., Nishinomiya, Japan), including depth, temperature, fresh-water EC, turbidity, chlorophyll-a, dissolved oxygen, PAR, pH, and ORP sensors, was immersed in the measuring box. Simultaneously, the main meteorological parameters were measured using a Vantage Pro2 weather station (Davis Instruments, Hayward, CA, USA). The parameters of the AHMS sensors are shown in Table 1.

Unlike the installation of the system on an anchored platform under the water, the organization of the flow-type system allowed us to avoid a complex underwater structure that keeps the system unmovable. The water intake was placed one meter above the bottom, which significantly reduces the possibility of sediments and other materials entering the pipeline. Additional advantages of the installation of the measuring equipment on the shore are as follows: (i) unlimited source of electricity; (ii) the ability to service measuring sensors at any time without the assistance of scuba divers; (iii) the ability to take water samples at any time for parallel laboratory research. At the pier of Limnological Institute, a closed cabin is arranged, where the equipment can be safely placed and two researchers can be comfortably accommodated (Figure 1 and Figure 2).

Rinko AAQ-177 is a water-quality profiler with a 100 m cable. It cannot record measured data, but only transmit them through the RS-485 interface to a personal computer (PC). In AHMS, the Rinko profiler is connected to a PC via RS-485 to the USB adapter supplied with the device. A software was written for PC, which examines the Rinko profiler every 10 s (can be configured) and saves the data to a local database controlled by PostgreSQL. The software also calculates ionic mineralization, based on an equation of dependence of the concentration of principal ionic components of Baikal water on the water conductivity [39], which reduces to zero hydrostatic pressure and to constant temperature [40]. The GPRS modem that provides the connection between the computer and the Internet is connected to the PC via USB. The main database controlled by PostgreSQL operates on the remote server of Limnological Institute. Both databases are synchronized in real-time using PostgreSQL’s built-in logical replication mechanism. This mechanism allows for a more granular control over data replication and data transfer security aspects. To implement this scheme in the local database for the data table from Rinko, a publication is created. A subscription to this publication is created in the main database, located on the server of Limnological Institute. Thereafter, changes, as they occur on the side of the publication, are transmitted to a subscriber in real-time. In the absence of communication, the data are accumulated in the local database and, after the restoration of the communication channel, are automatically transmitted.

**Figure 3:**
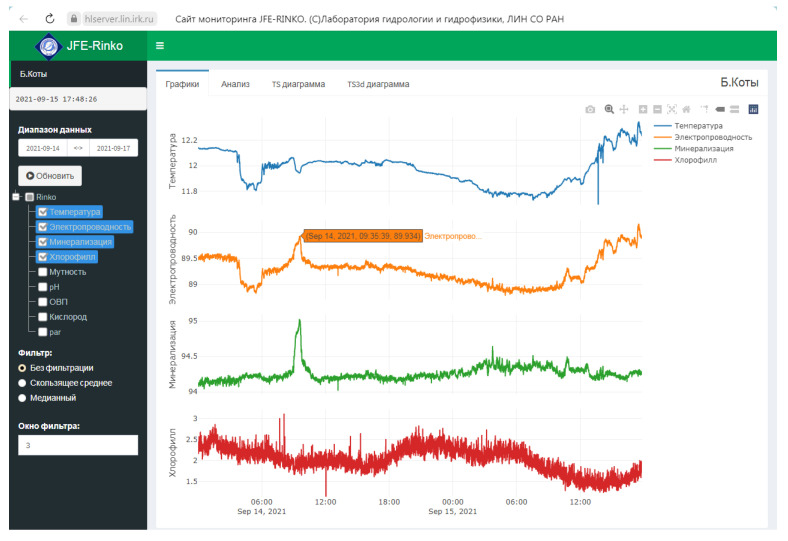
Web interface access to the AHMS data.

The remote server of Limnological Institute runs a web service [41] that provides access to the data of the monitoring system. Libraries of the high-level programming language, R [42] perform all procedures for fetching data from the database, mathematical processing, filtration, and analysis. The interface with the end user through the web service is carried out in the javascript language via the Shiny Server addon [43]. The service for accessing data via the web interface provides the following: (i) data-fetching for a certain time interval; (ii) displaying several parameters at the same time; (iii) visual data analysis (Figure 3).

The data acquisition PC is a laptop with an Intel processor, but a single-board computer, such as Beaglebone Black (processor: AM335x 1GHz ARM® Cortex-A8), with more modest specifications, is sufficient for these tasks. The software used to examine the profiler was written in a high-level programming language, pascal syntax. This software examines the Rinko profiler, converts the data into physical values based on calibration tables and records the data in the local database. Thereby, the software implements the main elements of data acquisition, data processing and data storage.

The monitoring system was first activated on 11 August 2020 and operated until 12 December 2020. However, with the onset of cold weather (air temperature dropped below −20 °C) and due to the risk that the water in the flow system would freeze, the monitoring system was deactivated. From December to late January, there is stormy weather at the lake, making it impossible to navigate and move from one settlement to another. The opportunity to move around the lake only reappears when the ice cover has completely formed, using wheeled or tracked vehicles. The second activation of the monitoring system was carried out on 16 April 2021, when the lake’s surface was still completely covered with ice. Complete ice breakup occurs in late April or early May.

We implemented an adaptive platform that we can independently customize for our needs by changing the frequency of data acquisition, changing the composition of sensors measuring water parameters and changing the presentation of, or conditions of access to, the measured parameters.

### 2.3. Verification of Data by Parallel Measurements in the Laboratory

The Rinko AAQ-177 profiler is supplied with calibration certificates for all installed sensors. As a rule, calibration values represent the polynomial coefficients. For some sensors, an additional temperature correction is introduced, for example, for a dissolved oxygen sensor or a pH sensor. However, the manufacturer of the Rinko profiler, the JFE Advantech Company, recommends performing an additional calibration on the standard solutions for the pH sensor and fast optical DO sensor immediately prior to measurement. Therefore, the records of these sensors were additionally verified by intercalibration with parallel laboratory analyses using water samples from the same site. This was carried out because we are especially interested in assessing the accuracy of the sensors in this measurement scheme, as well as comparing these methods with those traditionally used for Lake Baikal monitoring.

**Figure 4:**
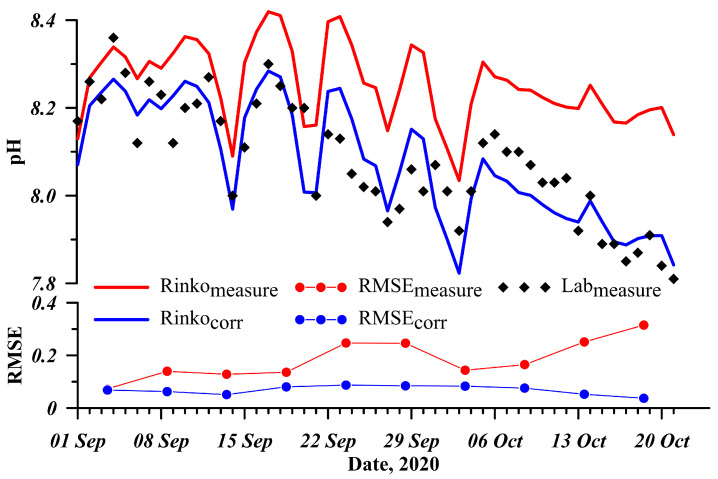
Correction of the pH sensor data based on the laboratory measurements.

A conductivity meter Expert-002-2-6-p (4 µS cm${}^{-1}$ accuracy) and pH-meter Expert-001 with 0.02 pH accuracy (both devices manufactured by Econix-Expert LLC, Moscow, Russia) were used to verify the measurements of electrical conductivity and pH, respectively. Winkler titration method was used to determine dissolved oxygen concentration with 0.3% precision. A comparison of electrical conductivity revealed no significant deviations exceeding the declared accuracy of the instruments, while measurements of dissolved oxygen showed a slight increase in oxygen saturation (by 2% to 5%) after its pumping into the measuring box. Apparently, this was due to strong mixing and contact with the atmosphere during inflow from the pipeline. We will try to organize more careful water inflow to the box during the installation of the next station. A rather strong drift in records over time was identified for the pH sensor. Therefore, we adopted a strategy that allowed us to correct the changes in the calibration characteristics by cross-calibration with the data obtained in the laboratory. A combined analysis of the data revealed that the sensor calibrations drift linearly with time, and this is relatively easy to correct using the data from single measurements with a laboratory pH-meter (Figure 4). RMSE decreased to <0.1 pH after the correction.

Figure 4 shows the data for the autumn season. During this period, there is a natural decrease in the photoactive solar radiation penetrating the water, which inhibits photosynthesis processes in the lake. This causes an increase in the concentration of carbon dioxide in water and a corresponding decrease in pH. This is a natural process, observed in lakes of temperate and sub-polar latitudes, including Lake Baikal (see e.g., [44]).

**Figure 5:**
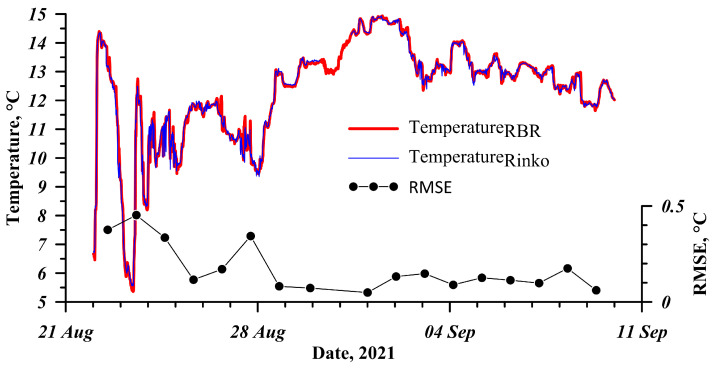
Comparison of temperature data measured by RBRSolo-T (red line) and AHMS (blue line).

When implementing a flow-type system to measure the parameters in the littoral zone of Lake Baikal, we were not sure that water would remain the same as long as it travelled from the sampling site to the measurement site. There are likely changes in parameters, such as temperature. With this connection, we studied the influence of the pipeline on temperature changes. To do this, we installed an RBRSolo-T temperature logger (RBR Ltd, Ottawa, ON, Canada) at the sampling site. The logger operated for 21 days; after this, we compared the temperature measured by the logger and that measured by AHMS (Figure 5). The measurements revealed insignificant changes that did not affect the data analysis, RMSE < 0.5 °C.

### 2.4. Maintenance of the Complex

Biofouling contamination is the main cause of a decrease in sensitivity and reliability in the measuring sensor records in limnological research [45]. The contamination occurs when suspended solids, bacteria, microalgae or larges organisms adhere to the surface of sensors. The rate of biofouling differs, depending on the temperature and season, and can range from one or two weeks to a month. Therefore, biofouling is currently a key factor that determines the duration of the operation of a device for measuring water quality, especially in systems of long-term and continuous monitoring.

At present, many antifouling strategies for sensors are available for use in water monitoring [45], including active systems (wiper/shutter/biocide injection-based systems, or ultrasonic/laser/UV irradiation techniques) or passive ones based on the application of various antifouling coatings, copper tapes, and copper-alloy sensor guards. Despite the wide range of antifouling strategies, to date, there is no effective, one-size-fits-all solution [46].

In AHMS, thanks to the flow-type system, the Rinko AAQ-177 profiler is easy to maintain. We only need to remove it from the measuring box and treat the sensors with distilled water and a special microfiber cloth or a brush. Such measures contribute to the stable operation of the sensors, preventing biofouling. Moreover, this is more convenient than if the monitoring system were installed at the buoy station.

### 2.5. Plausibility Tests

An automated quality control of obtained data was implemented in the server software in the form of plausibility tests that examine the range and variability of each parameter. Data that do not pass the test are not deleted but flagged as suspicious, with the raw data being stored in the database. A range test checks that every recorded observation falls within the reasonable minimum and maximum values. Thus, the water temperature cannot be below 0 °C or above 30 °C. The parameter thresholds used in the range tests are shown in Table 2.

Another plausibility test is used to ensure that changes in a time series of data are realistic over a given period. This test compares successive datapoints to determine if their difference exceeds a maximum threshold. This eliminates single outliers.

We estimate the percentage of data that are rejected after quality checks, which were different for each parameter. On average, about 0.4% of the data are rejected. The lowest percentage of the rejected data (about 0.01%) are for temperature and conductivity. The maximum percentage of rejections (about 2%) was for dissolved oxygen. Additionally, we estimated the percentage of completely lost data, which was 0.8% of all received data. Such cases were usually associated with power failures when the AHMS did not work at all.

**Figure 6:**
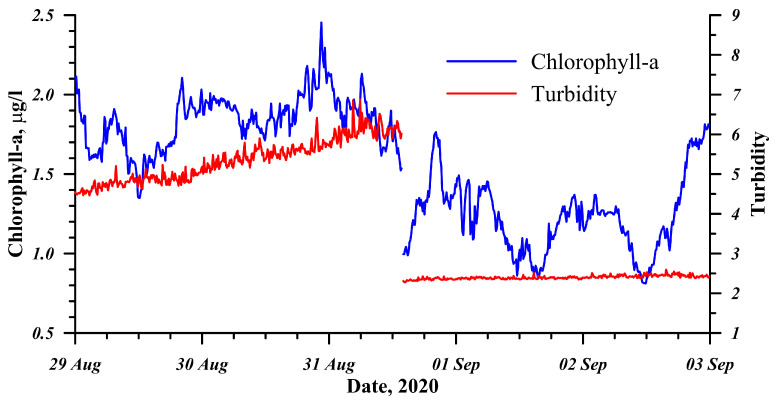
Process of biofouling of the sensors and the change in records after the cleaning.

As the data on the web interface were displayed with the R statistical environment, quality is controlled at the stage of displaying data for a specific user. This means that only the data that have passed the quality control are fetched from the database. This check will help to avoid erroneous data associated with single malfunctions or transmission/data storage errors. At the same time, there can be distorted data associated, for example, with the drift in the calibration characteristics of individual sensors. This primarily concerns the pH sensor, and the solution was described above, in Section 2.3. The data can be distorted during biofouling; this mainly concerns optical sensors such as chlorophyll-a fluorescence sensor and turbidity sensor. The consequence of biofouling is especially obvious after the sensors are cleaned, when their records return to normal (Figure 6). Water in Lake Baikal is rather clean, and its turbidity parameter usually does not exceed 5 FTU. Therefore, the signal from this sensor can be used in the procedure of the plausibility test as a biofouling indicator when its records exceed the threshold of maximum values. The automated data control applied to the server does not replace manual human verification but ensures data consistency and can process a large data flow, which is incompatible with the manual quality-control methods historically used by ecologists [47].

## 3. Discussion

The current technology development in the field of measuring water parameters (hydrophysical, hydrochemical and hydrooptical) provide the monitoring of water bodies with an unprecedented high frequency compared to conventional research. At the same time, the development of the automatic monitoring systems is coupled with a complex technology of the sensors, their maintenance, and the control of a large data volume. Our example should be considered a pilot experience of creating a monitoring station in the littoral zone of Lake Baikal, which mainly aims to obtain new, high-quality, quasi-continuous data series and training the operation of such automatic monitoring stations.

The first activation of the station from August to December 2020 allowed us to test the communication channel and data transmission algorithms as well as to control the integrity of data. In the same period, procedures were also performed to calibrate and compare the quality of the obtained data with the data from laboratory research. During the first three months, water for parallel laboratory analysis was sampled every four hours. This was the first experience in carrying out hydrophysical, hydrochemical and hyrdooptical research at Lake Baikal in automatic mode with data transmission to the server and access to these data in real time via the web service.

**Figure 7:**
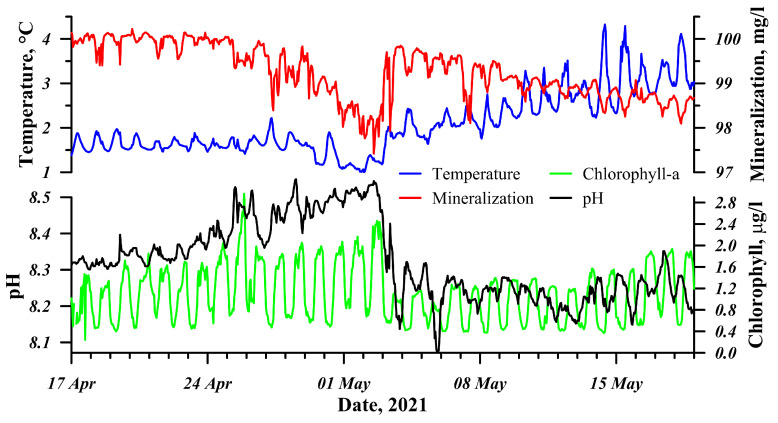
Time dynamics of various parameters from April to May 2021.

The second activation of the station allowed us to trace the processes occurring during the spring warming of the littoral zone of Lake Baikal in detail. Owing to the volumetric absorption of the gradually increasing solar irradiance, the water warms up and the ice cover melts. A warmer demineralizated surface water has a higher density and sinks to the bottom, generating convective mixing and decreasing the mineralization of the lower water layers. Figure 7 clearly shows the diurnal cycles of this process. The main factor affecting the change in the pH of the environment is the presence of carbon dioxide (CO${}_{2}$) in water, which gradually decreases in the spring under-ice period due to the rapid growth in microalgae that begin to receive more solar energy and nutrients resulting from the increased under-ice convection. This is evident from the increase in chlorophyll-a fluorescence and pH values in the second half of April (Figure 7), which was also revealed in other studies on Lake Baikal using classical methods [44]. Water flowing through the pipeline quickly enters the measuring site, and phytoplankton remains adapted to the current in situ illumination regime; hence, some recreation centres of photosystem 2 are inactived, underestimating the measurement results [48], which explains the daytime minimum chlorophyll-a values. To obtain the true values, it is necessary to recalculate them, considering the PAR intensity at the water sampling site according to the method developed for Lake Baikal [49]. In the evening on 2 May 2021, the wind began to intensify from the NW direction and, consequently, the ice cover was carried away from the shore for several kilometres. Simultaneously with this event, fresh, non-demineralizated water came from deeper layers of the lake, with a higher mineralization and lower pH (Figure 7). When the surface of the lake was released from the ice, more intensive water-heating began.

In addition, a sharp simultaneous change was periodically observed in the temperature and electrical conductivity of the analyzed water. As a rule, such changes occur due to wind action or changes in atmospheric pressure. For example, with the prolonged action of the “Verkhovik” wind (local name) blowing along the lake from the NE to the SW direction, the upper, more heated layer of water flows to the east coast. In turn, deep, cold and less mineralized water welled up from the pelagic zone on the west coast.

Based on this pilot experience, we can highlight the following key points for developing an effective system of automatic monitoring. (i) The data from the sensors should be processed by data quality control procedures to present reliable and consistent information about the quality of Baikal water. (ii) The automated control procedures should detect biological contamination of the sensors and signalize their treatment. (iii) The system can provide access to RAW data in real-time for all interested parties, but with a mandatory warning that the data may contain errors and must first be validated by experts. (iv) The development of an own-monitoring station opens up a wide range of opportunities for its modernization and reproduction. At present, we are planning to install a second comparative monitoring station at Lake Baikal in the Listvyanka settlement. This area is the most anthropogenically loaded coast of the entire lake. (v) It is advisable to create and develop a joint situation centre for collecting, storing and processing data, which would include: The Ministry of Natural Resources and Environment of the Russian Federation, the Federal Service for Hydrometeorology and Environmental Monitoring and the Russian Academy of Sciences. Combining data and analysis protocols, as well as overall open access, will offer a fresh perspective on the processes occurring in the coastal zone of Lake Baikal. Subsequently, we plan to monitor new parameters characterizing the dynamics of biogeocenosis, such as environmental DNA (eDNA) [50] or a network of acoustic radars that can automatically track stocks of commercial fish species [24,51].

## 4. Conclusions

The developed AHMS is an element of technology for automatic multiparameter monitoring stations that is yet to be introduced in the Baikal natural territory. This technology lies in the plane of the rational use of natural resources, which requires the development of systems and methods for the monitoring and control of water bodies, as well as for elaborating regulations in the field of water resource management. The set of sensors used in AHMS allows for basic water quality monitoring with high frequency and good accuracy, excluding continuous human engagement but, at the same time, requires the periodic cleaning of sensors. Due to the developed AHMS, a series of data on hydrophysical and hydrochemical parameters in spring (during ice breakup) and autumn seasons have been obtained when work with conventional sampling methods is extremely difficult. The general behaviour corresponds to that obtained in other studies, but, in our research, we managed to acquire detailed daily variations in parameters, which could be the subject of a separate study. The proposed concept of a measuring system can provide year-round measurements. The main condition for this is that there should be no power failures during the winter season, so the water pumping should not stop (in case of an emergency, a self-regulating heating cable can be mounted to thaw the pipeline). The developed conception can be sufficiently recommended for the implementation at water bodies in temperate and sub-polar latitudes. The implementation of such online environmental monitoring systems, combining sensors that measure parameters of the water environment, atmospheric pollutants, meteorological parameters, etc., can elucidate additional information about the causes of the ecological crisis at Lake Baikal. This is especially itrue f these systems autonomously operate for 24 h and transmit data to the situation centre for making operational decisions.

## Figures and Tables

**Table 1:** Accuracy and resolution of measured parameters of AHMS (parameters are marked with the following indices: 1—JFE Advantech, 2—Davis Instruments).

Sensor	Range	Resolution	Accuracy	Response Time
Temperature ${}^{1}$	−3–45 °C	0.001 °C	±0.01 °C (0 to 35 °C)	0.2 s
Conductivity ${}^{1}$	0–2000 µS cm${}^{-1}$	0.1 µS cm${}^{-1}$	±2 µS cm${}^{-1}$, (0 to 200 µS cm${}^{-1}$)	0.2 s
FTU ${}^{1}$	0–1000 FTU	0.03 FTU	±0.3 FTU or ±2%	0.2 s
Chlorophyll-a ${}^{1}$	0–400 µg L${}^{-1}$	0.01 µg L${}^{-1}$	±1% Full scale	0.2 s
DO ${}^{1}$	0–20 mg L${}^{-1}$, (0–200%)	0.001–0.004, µg L${}^{-1}$	±0.4 mg L${}^{-1}$, (±2% Full scale)	0.4 s
PAR ${}^{1}$	0–5000 µmol m${}^{-2}$s${}^{-1}$	0.1 µmol m${}^{-2}$s${}^{-1}$	±4%	0.2 s
pH ${}^{1}$	2–14 pH	0.01 pH	±0.2 pH	10 s
ORP ${}^{1}$	0–±1000 mV	0.1 mV	-	10 s
Air Pressure ${}^{2}$	420–820 mmHg	0.1 mmHg	1.3 mmHg	2.5 s
Air Humidity ${}^{2}$	0–100%	1%	0–100%	60 s
Rain ${}^{2}$	0–999.9 mm	0.25 mm	4%	20 s
Air Temperature ${}^{2}$	−40–+65 °C	0.1 °C	0.5 °C	10 s
Wind direction ${}^{2}$	0–360 ${}^{{}^{\circ}}$	1 ${}^{{}^{\circ}}$	0.3%	2.5 s
Wind speed ${}^{2}$	0.5–89 m/s	0.4 m/s	5%	2.5 s
Solar radiation ${}^{2}$	0–1800 W/m${}^{2}$	1 W/m${}^{2}$	5%	50 s
UV Index ${}^{2}$	0–16	0.1	8%	50 s

**Table 2:** Parameter thresholds used in the range tests.

Sensor	Min	Max	Unit
Water temperature	0	30	°C
Mineralization	90	98	mg/L
Turbidity	0	5	FTU
Chlorophyll-a	0	10	µg/L
Dissolved oxygen	50	150	%
pH	7.5	9.5	
ORP	100	400	mV

## Data Availability

Not applicable.
